# Respiratory drive: a journey from health to disease

**DOI:** 10.1186/s40560-024-00731-5

**Published:** 2024-04-22

**Authors:** Dimitrios Georgopoulos, Maria Bolaki, Vaia Stamatopoulou, Evangelia Akoumianaki

**Affiliations:** 1 Medical School, University of Crete, Heraklion, Crete Greece; 2 Department of Intensive Care Medicine, University Hospital of Heraklion, Heraklion, Crete Greece; 3 Department of Pulmonary Medicine, University Hospital of Heraklion, Heraklion , Crete Greece

**Keywords:** Ventilatory response to CO_2_, Metabolic hyperbola, Critically ill, Mechanical ventilation

## Abstract

Respiratory drive is defined as the intensity of respiratory centers output during the breath and is primarily affected by cortical and chemical feedback mechanisms. During the involuntary act of breathing, chemical feedback, primarily mediated through CO_2_, is the main determinant of respiratory drive. Respiratory drive travels through neural pathways to respiratory muscles, which execute the breathing process and generate inspiratory flow (inspiratory flow-generation pathway). In a healthy state, inspiratory flow-generation pathway is intact, and thus respiratory drive is satisfied by the rate of volume increase, expressed by mean inspiratory flow, which in turn determines tidal volume. In this review, we will explain the pathophysiology of altered respiratory drive by analyzing the respiratory centers response to arterial partial pressure of CO_2_ (PaCO_2_) changes. Both high and low respiratory drive have been associated with several adverse effects in critically ill patients. Hence, it is crucial to understand what alters the respiratory drive. Changes in respiratory drive can be explained by simultaneously considering the (1) ventilatory demands, as dictated by respiratory centers activity to CO_2_ (brain curve); (2) actual ventilatory response to CO_2_ (ventilation curve); and (3) metabolic hyperbola. During critical illness, multiple mechanisms affect the brain and ventilation curves, as well as metabolic hyperbola, leading to considerable alterations in respiratory drive. In critically ill patients the inspiratory flow-generation pathway is invariably compromised at various levels. Consequently, mean inspiratory flow and tidal volume do not correspond to respiratory drive, and at a given PaCO_2_, the actual ventilation is less than ventilatory demands, creating a dissociation between brain and ventilation curves. Since the metabolic hyperbola is one of the two variables that determine PaCO_2_ (the other being the ventilation curve), its upward or downward movements increase or decrease respiratory drive, respectively. Mechanical ventilation indirectly influences respiratory drive by modifying PaCO_2_ levels through alterations in various parameters of the ventilation curve and metabolic hyperbola. Understanding the diverse factors that modulate respiratory drive at the bedside could enhance clinical assessment and the management of both the patient and the ventilator.

**Supplementary Information:**

The online version contains supplementary material available at 10.1186/s40560-024-00731-5.

Respiratory drive, defined as the output of respiratory centers to respiratory muscles, is crucial in the management of critically ill patients. Recent data indicate that in these patients, both high and low respiratory drive may adversely affect patient outcomes through multiple pathways [1–5]. While the definition of respiratory drive may appear simple, without understanding its determinants and underlying pathophysiology, the term 'respiratory drive' often remains ambiguous. It is imperative to understand that in critically ill patients, ventilatory demands, as reflected by respiratory centers output (RCO) per minute (RCO/min), may deviate from actual minute ventilation (V’_E_) due to various reasons [2, 6]. Failure to consider this dissociation could hinder the recognition and management of high or low respiratory drive in critically ill patients. In this review, we aim to analyze the different aspects of respiratory drive to facilitate comprehension of the causes of high and low respiratory drive in spontaneously breathing or mechanically ventilated critically ill patients.

## Basic principles of control of breathing

1. Components of control of breathing system

The control of breathing system consists of three parts, a central control system in the brain (central mechanisms), a motor arm (effector) which executes the act of breathing, and a host of sensory mechanisms that convey information to the central controller (feedback mechanisms) [7–10].

For simplicity, the central controller can be considered as comprising two groups of neurons [7–10]: the brainstem group and the cerebral cortex group. The former, oversees the automatic (involuntary) aspect of breathing, and is divided into pneumotaxic, apneustic, and medullary centers. Each center includes a diverse group of neurons with specific roles in the breathing process. The cerebral cortex group is responsible both for voluntary (behavioral) and involuntary regulation of breathing.

The effector system consists of the pathways that transfer stimuli from the respiratory centers to neurons and thereafter to the respiratory muscles [2, 6]. The respiratory muscles involve the diaphragm, the main inspiratory muscle, as well as other inspiratory and expiratory muscles. Expiratory phase is usually passive at rest but may become active, characterized by expiratory muscles contraction, when high ventilatory demands exist [11]. Expiratory muscle contraction is common in critically ill patients [12].

The main feedback mechanisms of the control of breathing are: (1) chemical, (2) reflex, (3) mechanical, (4) metabolic rate, and (5) cortical [13]. Involuntary breathing is primarily regulated by chemical feedback and, to a much lesser extent, reflex feedback. Mechanical feedback, which involves changes in respiratory muscle pressure with volume (force–length) and flow (force–velocity) [14], is not relevant in critically ill patients since volume and flow are relatively small. Although the metabolic rate plays a key role in modulating the respiratory drive during exercise by linking CO_2_ production and elimination, in critically ill patients metabolic rate affects respiratory centers indirectly via alteration in metabolic hyperbola [2, 6]. Finally, the effects of cortical feedback are rather unpredictable, depending on the Intensive Care Unit (ICU) environment and patient factors (i.e., delirium). Furthermore, areas of the cortex (i.e., pre-inspiratory motor area) may be activated under certain circumstances for purposes that are largely unexplored [15].

- 2. Automatic act of breathing

**Fig. 1:**
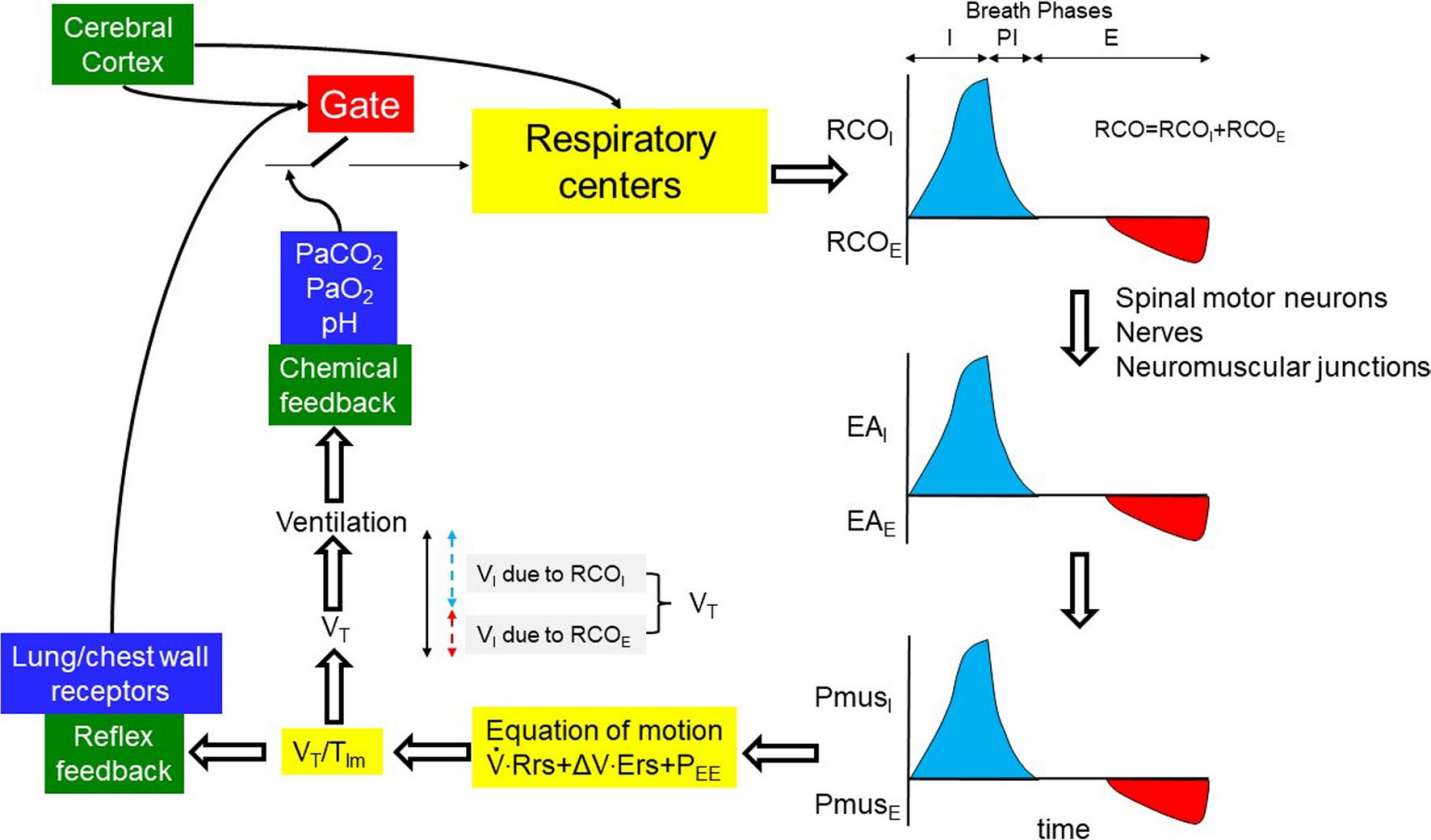
The inspiratory flow-generation pathway and the feedback mechanisms affecting it, in a normal subject during passive (no expiratory muscles activity) and active (expiratory muscles activity) expiration. For simplicity and demonstration purpose, RCO_I_ always begins when expiratory muscles activity ceases. Assuming that Pmus_E_ is able to lower lung volume below FRC (negative P_EE_), rapid relaxation of expiratory muscles (rapid decrease in Pmus_E_) passively generates inspiratory flow. When Pmus_E_ decreases to zero, FRC is reached. At this point Pmus_I_ increases and actively generates inspiratory flow. Notice, compared to passive expiration, the higher V_T_ with active expiration, which corresponds to higher RCO during the whole breath (respiratory drive). Gate: the effects of afferent signals (inputs) on respiratory centers vary, depending on the breath phases (inspiratory, post-inspiratory, expiratory); RCO: total respiratory centers output during the breath (respiratory drive); RCO_I_, RCO_E_: respiratory centers output to inspiratory and expiratory muscles, respectively; EA_I_, EA_E_: electrical activity of inspiratory and expiratory muscles, respectively; Pmus_I_, Pmus_E_: pressure generated by inspiratory and expiratory muscles, respectively; P_EE_: elastic recoil pressure of respiratory system at end-expiration (zero at FRC, and positive and negative at volume above and below FRC, respectively); Ers: respiratory system elastance; Rrs: respiratory system resistance; ΔV: volume above end-expiratory lung volume; V_T_: tidal volume; V_I_: inspired volume; blue areas: RCO_I_, EA_I_ and Pmus_I_; red areas: RCO_E_, EA_E_ and Pmus_E_; I, PI, E: inspiratory, post-inspiratory and expiratory phases, respectively; black double edges vertical arrow: V_T_; blue and red dashed double edges vertical arrows: contribution of inspiratory and expiratory muscle activity to V_T_

The automatic act of breathing entails the rhythmic activation of inspiratory and under certain circumstances expiratory muscles, via electrical bursts (outputs) from respiratory centers located in the medulla oblongata [9]. During this act of breathing the respiratory center receives inputs from various sources (mainly chemical and reflex feedback) that, through a complicated process, are translated into an output with an oscillatory pattern (Fig. 1). This output regulates the whole respiratory cycle which can functionally be divided into three phases: inspiratory, post-inspiratory, and expiratory. The duration of these three phases, although not always discrete, determines the timing of the breath and consequently the respiratory rate, whereas the intensity of the output is referred to as “respiratory drive”. The system employs “gating” to modulate the inputs, which means that the same tonic input may have a different effect on the respiratory centers, depending on the phase of the respiratory cycle [16]. Notably the neurons that control the breath timing (gate function) are different than these that control respiratory drive [17–19]. Cortical influences may interrupt this automatic process at any level [20, 21].

- 3. Chemical–reflex feedback mechanisms

Chemical feedback consists of the response of the respiratory centers to changes in arterial blood gases (PaO_2_, PaCO_2_) and pH [22]. PaCO_2_ is by far the strongest stimulus, acting on the respiratory centers either directly or indirectly, through the others [22]. A wide range of chemical feedback changes modify the respiratory drive, while the respiratory rate increases when the drive increases several folds above that of resting breathing [1, 23–25]. Reflex feedback, at least in adults, is much weaker and affects mainly the duration of the inspiratory and expiratory phases of the breath (i.e., Hering–Breuer reflex) [25–28].

- 4. Response to chemical stimuli

**Fig. 2:**
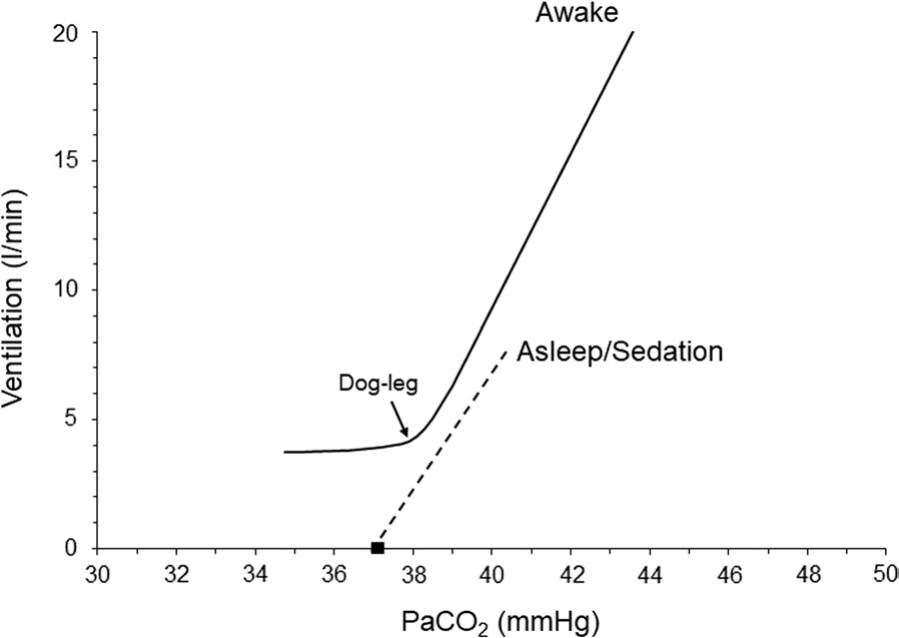
Ventilatory response to CO_2_ in a healthy individual. Notice the difference between the ventilatory response during wakefulness and sleep/sedation. Black square indicates the apneic threshold

We will particularly focus on the response to PaCO_2_ and PaO_2_. The normal response to hypercapnia involves a linear increase of V’_E_ as PaCO_2_ increases. The slope of this increase varies widely in healthy individuals, with an average value of 2–3 l/min/mmHg and a range of 0.6–8 l/min/mmHg [23, 29, 30]. The slope increases when there is hypoxemia or metabolic acidosis and decreases during sleep, sedation or metabolic alkalosis [22, 23, 31]. The hypocapnic response depends on the state of sleep and wakefulness. During wakefulness, the V’_E_–PaCO_2_ relationship continues to be linear as PaCO_2_ decreases. Nevertheless, the slope decreases rather abruptly, approaching zero at a certain PaCO_2_ level (dog-leg). This means that a minimum amount of V’_E_ (wakefulness drive to breath) is maintained at PaCO_2_ values well below this level [22]. During sleep or sedation, the PaCO_2_ to V’_E_ relationship remains linear until PaCO_2_ reaches a certain level where V’_E_ abruptly decreases to zero, resulting in apnea [32, 33] (Fig. 2).

Hypoxemia increases V’_E_, an effect that is modified by the PaCO_2_ and acid–base status [22, 23, 30]. Acute progressive isocapnic hypoxemia increases V’_E_ in a hyperbolic manner; V’_E_ remains almost unchanged as PaO_2_ drops to ≈ 60 mmHg, but at lower PaO_2_, it increases progressively with hypoxemia [34]. Although PaO_2_ is a weaker modulator of respiratory centers output (RCO) than PaCO_2_, it may significantly affect RCO and thus V’_E_ by modifying the response to PaCO_2_ [22, 23, 30].

## Respiratory drive and inspiratory flow-generation pathway

Respiratory centers output to inspiratory muscles travels from the brainstem and upper cervical spine neurons to the nuclei of inspiratory motoneurons (C3–C5 for the diaphragm) and determines the rate of phrenic nerve activity increase, which in turn, determines the rate of diaphragmatic muscle pressure increase. The latter determines the rate of volume increase and thus, depending on the respiratory rate, V’_E_ (Fig. 1) [2, 6]. At high ventilatory demands, the contraction of accessory inspiratory muscles supplements diaphragmatic pressure, further increasing the rate of volume expansion. Moreover, in this situation, the respiratory centers may stimulate expiratory muscle contraction. This could reduce the end-expiratory lung volume below functional residual capacity (FRC) [11]. Subsequent relaxation of expiratory muscles will generate inspiratory flow and contribute to final V_T_ [12]. Since the aim of expiratory muscle stimulation is to aid in V_T_ and alleviate the workload of inspiratory muscles [11], the term 'respiratory drive’ is defined as the total RCO to both inspiratory (RCO_I_) and expiratory (RCO_E_) muscles [6] (Fig. 1). The whole process described in a simplified manner, is collectively termed the ‘inspiratory flow-generation pathway’ [2].

When the inspiratory flow-generation pathway is intact, the resultant mean inspiratory flow, defined as the ratio between V_T_ and mechanical inflation time (T_Im_), aligns with that desired by the respiratory drive (RCO). In other words, the RCO per breath, corresponds to V_T_ and RCO/min to actual V’_E_. However, if there is any compromise in the integrity of the inspiratory flow-generation pathway, a dissociation occurs between the respiratory drive and the V_T_/T_Im_ [35]. Consequently, a given respiratory drive yields a smaller V_T_/T_Im_ and, all else being equal, lower V’_E_ (Fig. 1 and Additional file 1: Figs. S2 and S3). Although during the involuntary breathing the main determinant of respiratory drive is chemical feedback [2, 6], cortical inputs can highly affect respiratory drive when there is voluntary activity (pain, stress) [36]. However, at rest in the absence of voluntary activity, the cerebral cortex has an inhibitory influence on the respiratory center [37, 38]. This explains why patients with cortical lesions may exhibit high respiratory drive.

Since PaCO_2_ is the most important controller of the respiratory drive [2], it is important to briefly discuss what determines its value. At resting steady-state ventilation, PaCO_2_ is the point where the metabolic hyperbola intersects with the ventilatory response to CO_2_ curve [2, 29, 39]. The metabolic hyperbola graphically represents PaCO_2_ as a function of V’_E_, rate of CO_2_ production (V’CO_2_) and physiological dead space (V_D_) to V_T_ ratio as follows:

$${\text{PaCO}}_{2} = {\text{k}} \cdot {\text{V}}^{\phantom{a}'}{\text{CO}}_{2} /\left[ {{\text{V}}_{{\text{E}}}{\phantom{a}'} \cdot \left( {{1} - {\text{V}}_{{\text{D}}} /{\text{V}}_{{\text{T}}} } \right)} \right],$$

where k is constant (0.863) [39]. The ventilatory response to CO_2_ curve describes V’_E_ as a function of PaCO_2_ and depends on the (1) response of respiratory centers to CO_2_ and (2) integrity of inspiratory flow-generation pathway [2].

## Brain and ventilation curves

To elucidate the impact of defects in the inspiratory flow-generation pathway on respiratory drive, we have recently introduced the concepts of brain and ventilation curves [2]. The brain curve is a theoretical representation, outlining the desired V’_E_ set by the respiratory centers at a given PaCO_2_. In simpler terms, the brain curve is determined exclusively by the respiratory centers’ sensitivity to PaCO_2_, which is controlled by afferent information from peripheral and central chemoreceptors. The term 'ventilation curve' describes the actual V’_E_ produced by a given RCO/min. Unlike the brain curve, the ventilation curve is influenced not only by the respiratory centers’ sensitivity to PaCO_2_, but also by the integrity of the inspiratory flow-generation pathway (Fig. 1 and Additional file 1: Figs. S1, S2 and S3). As discussed above, the brain curve is mainly determined by respiratory drive over a wide range of PaCO_2_ [1].

**Fig. 3:**
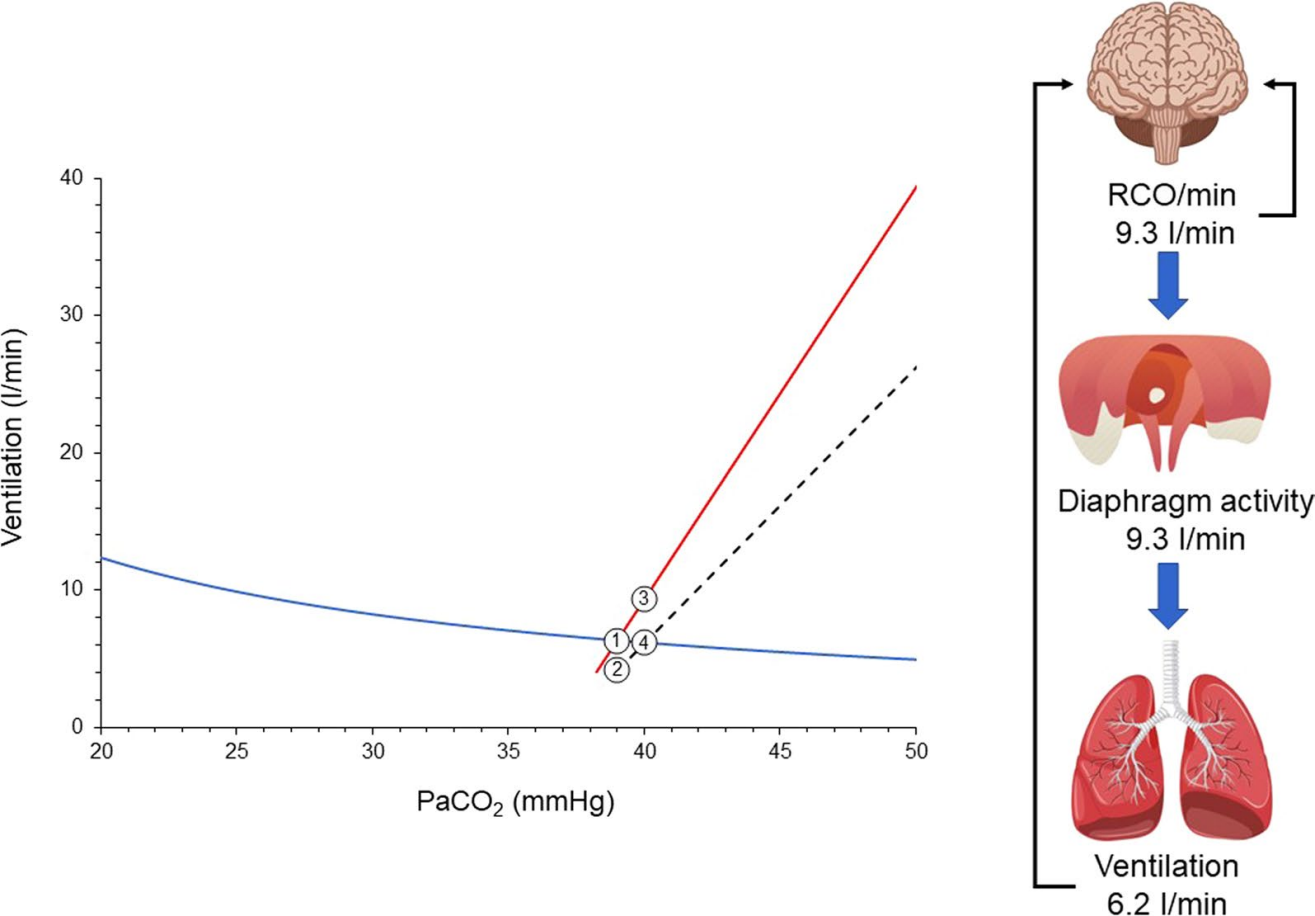
Brain curve (red line), ventilation curve (dashed black line), and metabolic hyperbola (blue line) in a spontaneously breathing patient with a disease affecting the inspiratory flow-generation pathway at the equation of motion level [e.g., restrictive disease (↑Ers), obstructive disease (↑Rrs), dynamic hyperinflation (↑P_EE_)]. Similar effects are anticipated if the integrity is compromised at higher levels of the inspiratory flow-generation pathway. PaCO_2_ desired by the brain is 39 mmHg and this corresponds to RCO/min of 6.3 l/min (point 1). In an intact inspiratory flow-generation pathway, the brain and ventilation curves would coincide, resulting in an actual PaCO_2_ of 39 mmHg. For simplicity, let us assume that the disease acutely compromises the integrity of inspiratory flow-generation pathway and as a result the ventilation curve is moved to the right with a downward slope. Brain curve and metabolic hyperbola are kept constant. Consequently, the RCO/min corresponding to 6.3 l/min decreases actual ventilation to 4.2 l/min (point 2). This decrease in ventilation triggers a gradual rise in PaCO_2_, stimulating the respiratory centers. RCO/min progressively increases (mainly due to changes in respiratory drive, RCO per breath) along the brain curve in response to the elevated PaCO_2_. As RCO/min increases, so does actual ventilation along the ventilation curve. A steady state is reached when RCO/min (point 3) yields actual ventilation at the intersection of the ventilation curve and metabolic hyperbola (point 4). At this point, PaCO_2_ stabilizes at 40 mmHg, and respiratory drive, RCO/min, and ventilation cease increasing as the CO_2_ stimulus remains constant. Despite ventilatory demands of 9.3 l/min, only 6.2 l/min are met, resulting in a deficit of 3.1 l/min. The respiratory centers activity and ventilatory output are projected to forebrain via the corollary discharge pathway (re-afferent traffic, black arrows) and create the sense of dyspnea. Given the relatively low RCO/min and unmet demands, this patient is unlikely to experience dyspnea, particularly during resting conditions

When the inspiratory flow-generation pathway is intact, the brain and ventilation curves are identical. However, if the integrity of the pathway is compromised, the ventilation curve deviates (is shifted down and to the right) from the brain curve (Fig. 3). As a result, the metabolic hyperbola and ventilation curve intersect at a higher level of PaCO_2_ than that desired by the brain (the PaCO_2_ that would result from the intersection of the brain curve and metabolic hyperbola) [2, 6]. Elevated PaCO_2_ stimulates the respiratory centers, prompting an increase primarily in their output per breath (RCO, respiratory drive) and, to a lesser extent, in respiratory rate [1]. Consequently, factors that modify the positioning and inclination of the ventilation curve, the brain curve, and/or the metabolic hyperbola influence the respiratory drive [2, 6].

## Causes of high and low respiratory drive

High or low respiratory drive results from alterations in the (1) brain curve, (2) ventilation curve and (3) metabolic hyperbola. In critically ill patients usually high or low respiratory drive is the result of combined changes in these three curves. Brain curve is altered by PaO_2_ changes, acid–base disturbances, neurotransmitters affecting the brain stem and stimulation of various receptors mainly located in the respiratory system [30, 40–43]. In general, hypoxemia, metabolic acidosis, and lung/chest wall receptors stimulations concurrently shift the brain curve leftwards and upwards, whereas hyperoxemia, metabolic alkalosis, and sleep or sedation shift it rightwards and downwards [30, 44–46]. In critically ill patients breathing spontaneously, the inspiratory flow-generation pathway is impaired (Table 1), shifting the ventilation curve to the right and downwards. This causes a consistent deviation of the ventilation curve from the brain curve (Fig. 3). As a result, actual PaCO_2_ is higher than that desired by the respiratory centers, which respond by increasing RCO/min along the brain curve. When RCO/min results in an actual V’_E_ at the intersection of the ventilation curve and the metabolic hyperbola, a steady state occurs. PaCO_2_ stabilizes and RCO/min and V’_E_ do not increase further. Although the ventilatory demands are not met, the RCO/min does not increase further because the CO_2_ stimulus remains constant (Fig. 3).

**Table 1:** Common causes of defects in inspiratory flow-generation pathway in critically ill patients

Level	Causes
Motor neurons	Trauma/ALS
Phrenic nerve	Critical illness polyneuropathy
Neuromuscular junction	NMBA/myasthenia gravis/poisoning
Diaphragm	Myotrauma
Equation of motion	
↑Rrs	Obstructive diseases (asthma/COPD)
↑Ers	Restrictive diseases (ARDS)
↑P_EE_	Dynamic hyperinflation

ALS: amyotrophic lateral sclerosis; NMBA: neuromuscular blocking agents; Rs: resistance of respiratory system; Ers: elastance of respiratory system; P_EE_: elastic recoil pressure of respiratory system at the end of expiration; COPD: chronic obstructive pulmonary disease; ARDS: acute respiratory distress syndrome

Mechanical ventilation may shift the ventilation curve either to the left or to the right of the brain curve, depending on the level of assist provided. The slope of the curve is heavily regulated by the mode of support [2]. Therefore, during mechanical ventilation, the theoretical PaCO_2_, determined by the intersection between metabolic hyperbola and brain curve, may be higher or lower than the actual PaCO_2_, causing a decrease or increase in respiratory drive, respectively. The decrease in respiratory drive during mechanical ventilation, resulting from leftward shift of the ventilation curve, is common and can induce unstable breathing [2] (see below). This is infrequent in unsupported breathing, occurring mainly in specific diseases or circumstances (congestive heart failure, sleep apnea syndrome, high altitude) [47–49].

The metabolic hyperbola determines both the desired PaCO_2_ and the actual PaCO_2_ levels. Consequently, its upward or downward shifts significantly impact these PaCO_2_ levels, thereby affecting the respiratory drive. Increased V’CO_2_ and V_D_/V_T_ ratios shift the metabolic hyperbola upward, whereas decreases in these variables shift it downward [29]. In critically ill patients, changes in V’CO_2_ are induced by alterations in metabolic rate, which can be influenced by the disease itself (e.g., sepsis), body temperature, or vigorous respiratory efforts [50–52]. Ventilator settings, breathing patterns, V’/Q’ inequalities, right-to-left shunt, and modifications in dead space influence V_D_/V_T_ [39]. Notably, a rapid, shallow breathing pattern secondary to delirium or panic reactions may cause an upward shift in the metabolic hyperbola due to an increase in V_D_/V_T_.

## Respiratory drive—from health to disease

To better understand the interaction between metabolic hyperbola and brain and ventilation curves let us follow the respiratory drive of an adult human from health to disease.

1. Health

**Fig. 4:**
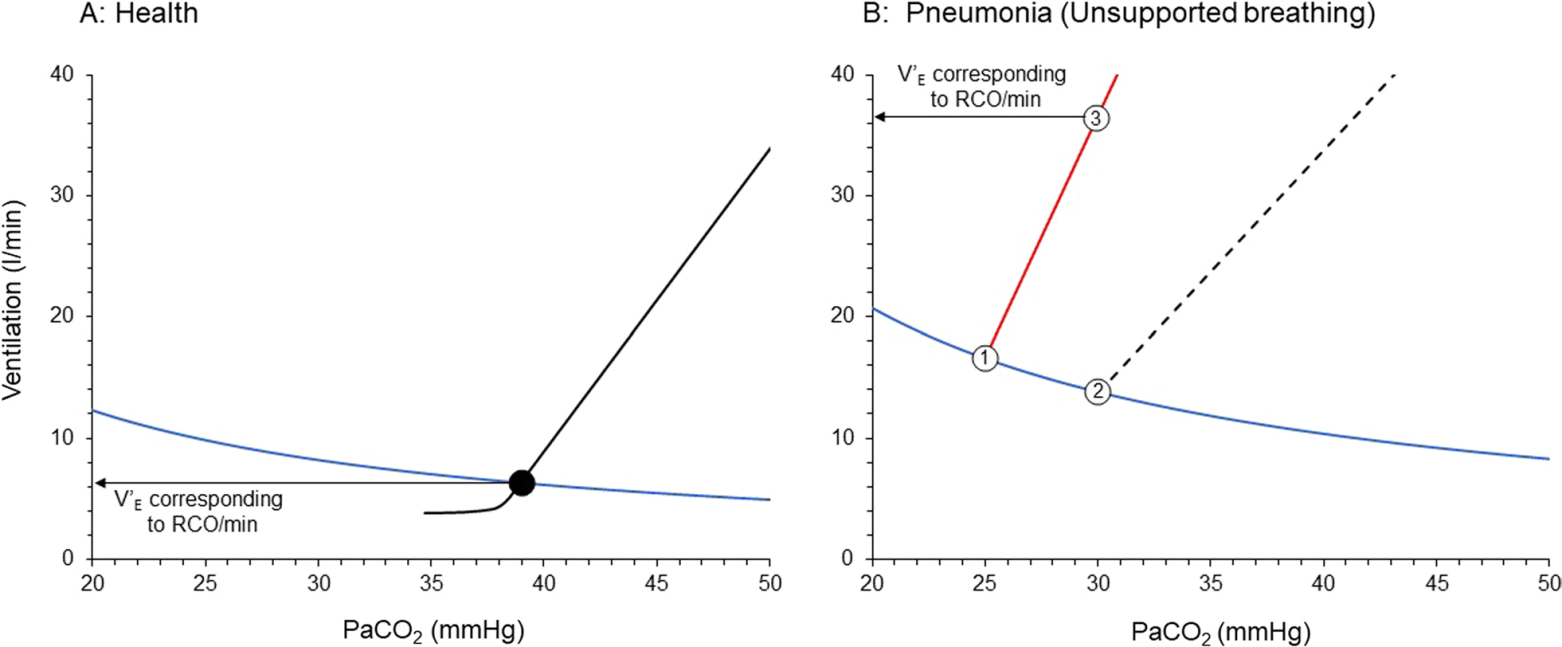
Brain and ventilation curves and metabolic hyperbola in a healthy subject (**A**) and when this individual suffers from pneumonia due to COVID-19 (**B**). **A** Health. Notice that brain and ventilation curves are similar (black lines) and thus the RCO/min corresponds to actual PaCO_2_ and ventilation, set by the intersection point (black circle) between ventilation curve and metabolic hyperbola (blue line). **B** This human develops severe pneumonia due to COVID-19, causing increased V’CO_2_ and V_D_/V_T_ which move the metabolic hyperbola upward. The concomitant hypoxemia and metabolic acidosis shift the brain curve to the left and increases its slope (red line). Due to increased respiratory system elastance, a given RCO/min results in a lower ventilation and thus, the slope of the ventilation curve (dashed black line) is shifted downward. A dissociation between the ventilation curve and brain curve occurs. The desired PaCO_2_ is 25 mmHg (point 1) and at this level of PaCO_2_ RCO/min corresponds to 16.6 l/min. The actual PaCO_2_ is 30 mmHg (point 2) and ventilation 13.8 l/min. PaCO_2_ of 30 mmHg represents hypercapnia for respiratory centers which increase their activity along the brain curve. Respiratory activity stabilizes to a level corresponding to 36.6 l/min (point 3). Unmet ventilatory demands are 22.8 l/min. RCO/min: respiratory centers output per minute

In a healthy individual the inspiratory flow-generation pathway is intact and thus the brain curve and ventilation curve are identical, over a wide range of PaCO_2_. Assuming that in a healthy adult (1) V’CO_2_ and V_D_/V_T_ are normal, 200 ml/min and 0.3, respectively; (2) the ventilatory response to CO_2_ is 2.5 l/min/mmHg; and (3) the intersection point between the metabolic hyperbola and ventilation curve is at PaCO_2_ of 39 mmHg (eupneic PaCO_2_), the resulting actual V’_E_ is 6.3 l/min. Since the brain and ventilation curves are identical, the RCO/min corresponds to 6.3 l/min, identical to the actual V’_E_ (Fig. 4A). Because there is no deficit between the ventilatory demands, as reflected by RCO/min, and actual V’_E_, the automatic act of breathing remains unnoticed by the forebrain [53, 54].

Notably, even in healthy individuals, extreme hyperventilation may cause a deviation between brain and ventilation curves, due to dynamic hyperinflation and/or increases in respiratory system elastance as high tidal volumes approach the total lung capacity towards the end of inspiration [6].

- 2. Disease

Let us consider a scenario where this adult develops pneumonia due to COVID-19. The patient is febrile (39 °C) and visits the Emergency Department of the regional Hospital, reporting breathing difficulties (dyspnea). Clinical examination reveals tachycardia and signs of increased work of breathing, while arterial blood gases show hypoxemia (PaO_2_ 45 mmHg on 21% F_I_O_2_) and hypocapnia (PaCO_2_ 30 mmHg). Acid–base balance evaluation demonstrates high anion gap metabolic acidosis. Chest X-rays are remarkable for diffuse opacities with loss of volume in the dependent lung regions. The patient has PaO_2_/F_I_O_2_ < 300 mmHg on high-flow nasal oxygen and meets acute respiratory distress syndrome (ARDS) criteria [55].

Let us consider, the expected alteration in brain curve, ventilation curve and metabolic hyperbola in this patient. This approach was recently used to explain the pathophysiology of dyspnea on exertion in patients with pulmonary hypertension [6].

I.  Unsupported spontaneous breathing

The inspiratory flow-generation pathway will be altered because of ARDS that induced a considerable increase in respiratory system elastance and slight increase in airway resistance [56, 57]. Therefore, compared to healthy status, a given RCO (respiratory drive) results in a lower V_T_. Hence, at a given respiratory rate, the ventilation curve is shifted to the right with a decreased slope, causing deviation between brain and ventilation curve; the actual PaCO_2_ is now higher than the theoretical PaCO_2_.

The brain curve shifts to the left due to increased respiratory centers sensitivity to CO_2_. The higher CO_2_ sensitivity is attributed to (1) hypoxemia, (2) metabolic acidosis and stimulation of lung receptors by the inflammatory process and lung mechanics deterioration [23, 30, 40, 41]. The resulting “theoretical” PaCO_2_, the one determined by the intersection of the brain curve and the metabolic hyperbola, will be much lower than in healthy state. Hence, even if the actual PaCO_2_ will be low, and the patient will have hypocapnia, it will be interpreted by the respiratory centers as “hypercapnia” when the desired PaCO_2_ is lower.

The metabolic hyperbola is shifted upward for two reasons. Firstly, V’CO_2_ increases due to pneumonia, fever and excessive work of breathing [50–52, 58]. Secondly, V_D_/V_T_ is increased due to V’/Q’ inequalities (high and low), the presence of right-to-left shunt (atelectasis) and in situ thrombosis in small pulmonary arteries and capillaries vessels, all of which increase the physiological dead space [39].

Figure 4B shows simulation of brain and ventilation curves and metabolic hyperbola, taking into consideration the pathology of this patient.

The brain curve is constructed assuming that the sensitivity of the respiratory centers increases by 60% from that in a healthy state, reaching 4 l/min/mmHg. The theoretical intersection point between the metabolic hyperbola and the brain curve is set at 25 mmHg, which is 5 mmHg lower than the actual PaCO_2_. The metabolic hyperbola is shifted upwards due to a 20% increase in V’CO_2_ to 240 ml/min and a 67% increase in V_D_/V_T_ to 0.5. Finally, the slope of the ventilation curve, mainly due to an increase in respiratory system elastance, decreases to 2 l/min/mmHg, resulting in a considerable deviation between the brain and ventilation curves. At PaCO_2_ of 30 mmHg, actual ventilation is 13.8 l/min, while at this level of PaCO_2_ the brain curve dictates that RCO/min corresponds to 36.6 l/min, a 22.8 l/min deficit between the ventilatory demands and actual V’_E_. This high RCO/min is mainly due to an increase in RCO (respiratory drive) which augments respiratory muscles (inspiratory and expiratory) activity per breath. Respiratory rate may increase when respiratory drive is 3–5 times higher than the baseline [1]. The high respiratory centers activity and the unmet ventilatory demands are projected via the corollary discharge pathway to the forebrain and create the subjective symptom of dyspnea [53, 54].

### Consequences of high respiratory drive

The consequences of the high respiratory drive in this patient are numerous. Firstly, the high respiratory muscles activity per breath places the patient at risk of self-inflicted lung injury (P-SILI) [3]. Indeed, patients with a high respiratory drive may experience increased regional stress and strain in dependent lung regions due to the pendelluft phenomenon, characterized, early in inspiration, by the movement of air within the lung from nondependent to dependent regions without a change in V_T_ [59]. Secondly, because of high elastance the transpulmonary driving pressure is high, contributing to lung injury [60]. Thirdly, the intense contraction of the diaphragm is associated with diaphragm damage [4, 61]. This should be of great concern in this patient, as increased expression of genes involved in fibrosis and histological evidence for the development of fibrosis in the diaphragm have been reported in COVID-19 ICU patients [62]. Finally, the vigorous inspiratory efforts that lead to excessive negative esophageal pressure swings increase the trans-capillary pressure of pulmonary vessels and the afterload of the left ventricle, both of which are risk factors for increased capillary leak into the alveoli [63, 64].

### Estimation of respiratory drive

How can we estimate the respiratory drive in this patient? Although the respiratory drive cannot be measured directly in humans, it can be indirectly estimated via various indices. Since the inspiratory flow-generation pathway is compromised at the level of equation of motion, the V_T_/T_Im_ no longer corresponds to respiratory drive and thus cannot be used as an index of it [2]. Provided that the inspiratory flow-generation pathway is intact up to the level of respiratory muscles, in order to estimate respiratory drive, we must obtain indices of respiratory motor output, such as electrical activity of the diaphragm (EAdi), trans-diaphragmatic pressure (Pdi), respiratory muscle pressure (Pmus), airway occlusion pressure (P0.1) and diaphragm thickening during inspiration (quantified by the thickening fraction, TFdi) [2, 5, 65] (Table 2). However, obtaining these indices requires expertise, and measuring them presents some challenges in spontaneously breathing patients with acute respiratory failure and distress. Therefore, clinical criteria of respiratory distress must be used to estimate the respiratory drive in this patient. It follows that the physical examination is of paramount importance in respiratory drive evaluation. Clinical signs of respiratory distress, such as hypertension, diaphoresis, tachycardia, accessory inspiratory (sternocleidomastoid, scalenes, external intercostals) and expiratory muscles (abdominals) contraction, nose flaring and intercostal retraction serve as reliable markers of high respiratory drive (Table 2). Despite the common belief that the respiratory rate is a sensitive index or respiratory drive, the latter should be markedly increased (3–5 times) before the former can change [1].

II. Mechanical ventilation

**Table 2:** Indices of potential injurious low and high respiratory drive in critically ill patients

Indices	Low drive	High drive
Clinical signs/symptoms	Apneas^a^	Respiratory distress^b^
ΔPdi	< 3 cmH_2_O	≥ 12 cmH_2_O
Pmus_sw_^c^	< 3 cmH_2_O	≥ 15 cmH_2_O
ΔPes	> − 3 cmH_2_O	< − 8 cmH_2_O
PTP/min^c^	< 50 cmH_2_O*s/min	> 200 cmH_2_O*s/min
P0.1	< 1–1.5 cmH_2_O	> 3.5–4 cmH_2_O
P_occl_^d^	> − 4 cmH_2_O cmH_2_O	≤ − 20 cmH_2_O

ΔPdi: trans-diaphragmatic pressure increase during inspiration; Pmus_sw_: pressure swings of respiratory muscles (inspiratory and expiratory) during the breath; ΔPes: negative change in esophageal pressure from the end-expiratory level; PTP/min: esophageal pressure (Pes)–time product per minute (PTP/min), calculated as the difference between Pes and the chest wall elastic recoil pressure during inspiration

^a^The only reliable clinical sign of low drive in a mechanically ventilated patient is the occurrence of repetitive apneas (Cheyne–Stokes breathing) as a result of over-assist

^b^Clinical signs and symptoms indicating respiratory distress are numerous, including accessory inspiratory muscles use, expiratory muscles contraction, diaphoresis, tachycardia, nose flaring, intercostal retraction and dyspnea

^c^Calculation of Pmus_sw_ and PTP/min necessitates measurement of chest wall elastance (passive conditions, unreliable in patients with active breathing)

^d^When P_occl_ is multiplied by − 0.75 and − 0.66, a gross estimate of Pmus_sw_ and ΔPes from un-occluded tidal breaths can be obtained, respectively

The patient is admitted to ICU and although high-flow nasal O_2_ therapy was applied, hypoxemia (SaO_2_ 85–88%) and respiratory distress continued. A decision to intubate was made. The patient was sedated and placed on volume control mode. Since vigorous respiratory efforts were not completely eliminated due to high respiratory drive [66, 67], neuromuscular blocking agents were administered. The elimination of respiratory efforts combined with the decrease in body temperature using non steroid anti-inflammatory agents, decreased V’CO_2_ production to 200 ml/min and moved metabolic hyperbola downwards. However, despite using a humidifier to prevent the decrease in dead space caused by heat and moisture exchange filters [68], V_D_/V_T_ remained high, resulting in minimal downward movement of the metabolic hyperbola. Lung protective strategy was applied, hypoxemia was corrected, while PaCO_2_ was maintained at 40 mmHg.

The next day paralysis was interrupted while sedation gradually decreased and stopped. When inspiratory efforts were resumed a premature decision to place the patient on pressure support (PS) was made, assuming that the high respiratory drive can be controlled by assisted mechanical ventilation. Nevertheless, the common belief that mechanical ventilation decreases respiratory drive due to unloading is disputed. Studies have shown that mechanical ventilation reduces respiratory drive indirectly by altering chemical feedback, primarily PaCO_2_ levels [25, 69]. Respiratory drive consistently follows chemical feedback, whether with or without mechanical ventilation. Therefore, during assisted mechanical ventilation, an intellectual theoretical assessment of brain and ventilation curves, and metabolic hyperbola, remains essential for understanding abnormalities in respiratory drive.

**Fig. 5:**
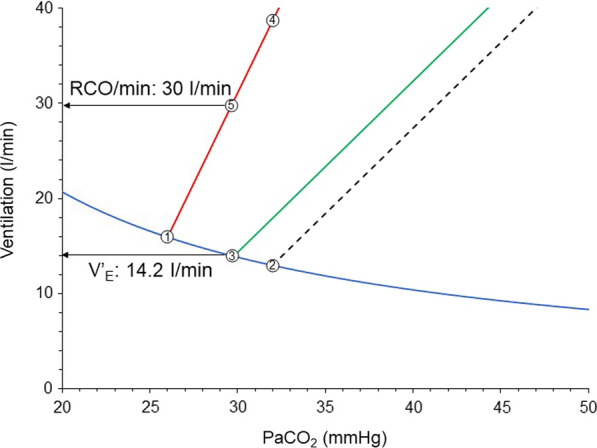
Brain curve (red line), unsupported (dashed black line) and supported with PS (green line) ventilation curves early in the course of critical illness of the patient of Fig. 4. Point 1: desired PaCO_2_ by respiratory centers; Point 2: theoretical PaCO_2_ during unsupported spontaneous breathing; Point 3: actual PaCO_2_ with PS during stable breathing (steady state); Point 4: RCO/min corresponding to desired V’_E_ with unsupported spontaneous breathing; Point 5: RCO/min corresponding to desired V’_E_ with PS ventilation; Notice the unmet demands without (difference in ventilation between points 2 and 4), and with PS (difference in ventilation between points 3 and 5). PS: pressure support; RCO/min: respiratory centers output per min; V’E: minute ventilation

The patient continues to exhibit high anion gap metabolic acidosis. Although brain curve is slightly shifted to the right due to correction of hypoxemia, its slope continues to be high, since stimulation of receptors and metabolic acidosis are maintained [41, 44]. Although the desired PaCO_2_ by the respiratory centers increased slightly, the respiratory system mechanics were not improved and, therefore, the deviation between the brain and ventilation curves remains considerable (Fig. 5). At a given constant respiratory rate PS shifts the unsupported ventilation curve parallel to the left [2]. The actual PaCO_2_ is 29.9 mmHg, 3.8 mmHg higher than the desired PaCO_2_ and actual V’_E_ is 14.2 l/min. Because the actual PaCO_2_ is higher than the desired, RCO/min increases along the brain curve to 30 l/min. Provided that respiratory muscles are not compromised, the activity of respiratory muscles also correspond to 30 l/min. This high activity of respiratory muscles is a risk factor for P-SILI and patient–ventilator dyssynchrony [3, 70]. Additionally, at this level of respiratory drive there is recruitment of expiratory muscles which contract and decrease end-expiratory lung volume below that determined by PEEP [12]. This may potentially cause further lung injury (atelectrauma), derecruitment, and gas exchange abnormalities. Deterioration of respiratory system mechanics and gas exchange abnormalities move the brain curve to the left and metabolic hyperbola upwards [2].

### Estimation of respiratory drive during mechanical ventilation

How can we estimate the respiratory drive in this patient? In mechanically ventilated patients respiratory drive can be quantitated using indices of motor output as described above. These indices, contrary to spontaneous breathing patients, can be obtained relatively easily [5, 65]. Yet again, it is important to recognize that the presence of a disease that affects the inspiratory flow–generation pathway at or before the anatomical site of measurement always leads to underestimation of the respiratory drive. Respiratory muscles weakness is common in critically ill patients. Nevertheless, despite this limitation, indices of respiratory motor output may provide to the physician information for injurious high drive and assist the decision-making process (Table 2). Values for Pdi increase during the inspiratory phase (ΔPdi) ≥ 12 cmH_2_O and respiratory muscle swings during the breath (Pmus_sw_) ≥ 15 cmH_2_O are associated with high drive which may be injurious, whereas driving transpulmonary pressure (ΔP_lung_) ≥ 12 cmH_2_O and transpulmonary pressure swings (Plung_sw_) ≥ 20 cmH_2_O indicate high lung stress and strain [4]. P0.1 higher than 4 cmH_2_O, easily measured in all ventilators, has an excellent accuracy to detect high effort per breath [71]. It has been shown recently that P0.1 higher than 3.5 cmH_2_O is associated with increased mortality [72]. The absolute drop in Paw during a whole breath occlusion correlates also with pleural and respiratory muscles pressures changes during the un-occluded tidal breaths [73, 74], but its interpretation might be heavily affected by cortical feedback in awake patient and does not provide more information than P0.1. Finally, TFdi > 30% is an index of intense diaphragm contraction [75].

In this patient, due to deviation between the supported ventilation curve and brain curve unmet ventilatory demands are 15.8 l/min (30.0–14.2). For this reason, the patient exhibits signs of respiratory distress, which may force the clinicians to increase the level of assist. Since in this patient the desired PaCO_2_ is 26 mmHg the PS level should considerably increase to achieve this value, resulting in excessive mechanical power applied on the lung [76] and increased afterload of the right heart [77]. The latter is attributed to high transpulmonary pressure which increases the pulmonary vascular resistance by creating zone II and I conditions in pulmonary circulation, potentially leading to acute cor pulmonale [78]. Therefore, this strategy increases the risk of lung injury and right heart dysfunction.

The indices of respiratory motor output and clinical examination, including dyspnea assessment [79, 80], indicate injurious high drive (Table 2) and thus the patient was placed back to protective mechanical ventilation. Another attempt for fully assisted modes should be considered when the causes of alterations in brain curve, ventilation curve and metabolic hyperbola will be addressed. It is important to notice that during protective mechanical ventilation, if it is possible, complete inactivity of inspiratory muscles should be avoided in order to reduce the risk of atrophy [4].

After 3 days the patient meets criteria for assisted mode. Respiratory system mechanics and gas exchange abnormalities have been improved, indicating partial resolution of ARDS, while high anion gap metabolic acidosis has been resolved. The patient exhibits metabolic alkalosis mainly due to hypoalbuminemia.

**Fig. 6:**
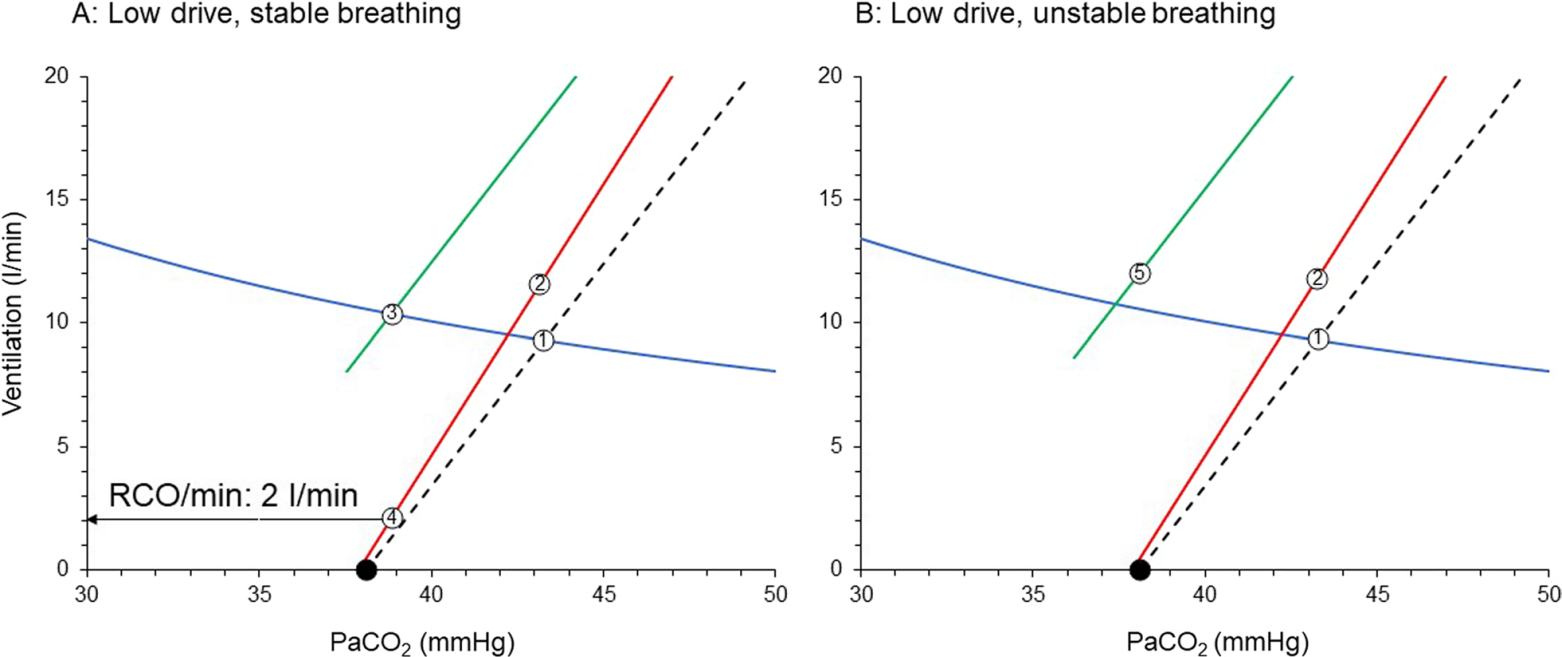
Brain curve (red line), unsupported (dashed black line) and supported with PS (green line) ventilation curves, relatively late in the course of critical illness of the patient of Fig. 5. **A** High PS, stable breathing. **B** Unstable breathing with increasing PS. Point 1: PaCO_2_ during unsupported spontaneous breathing; Point 2: RCO/min corresponding to desired V’_E_ with unsupported spontaneous breathing; Point 3: actual PaCO_2_ with PS during stable breathing (stable ventilation); Point 4: RCO/min corresponding to desired V’_E_ with PS ventilation (stable ventilation); closed circles: apneic threshold; Point 5: Actual V’_E_ that results in apnea; Notice that with PS ventilation curve is shifted to the left of brain curve. See text for further explanation. PS: pressure support; RCO/min: respiratory centers output per min; V’_E_: minute ventilation

The patient is placed on PS and a relatively high level of assist was used. At the same time a light sedation strategy is applied and if needed, an analgetic opioid is administered. Sedation, opioid, metabolic alkalosis and resolution of ARDS decrease considerably the sensitivity to CO_2_ and shifts the brain curve to the right with a downward slope [31, 43, 45]. This rightward shift of the brain curve combined with high assist level [2], place the supported ventilation curve to the left of the brain curve (Fig. 6A). Actual PaCO_2_ and V’_E_ are 39 mmHg and 9.7 l/min, respectively. The desired PaCO_2_ by respiratory centers is 42 mmHg and RCO/min at this PaCO_2_ corresponds to 9.0 l/min. However, since the actual PaCO_2_ is below 42 mmHg, the RCO/min decreases to that dictated by the PaCO_2_ of 39 mmHg, which is 2.0 l/min. The respiratory drive is so low that the patient relaxes the diaphragm soon after triggering. This can be confirmed by indices of respiratory motor output as described above and TFdi. Values of ΔPdi and ΔPmus_sw_ ≤ 3 cmH_2_O, P0.1 < 1.5 cmH_2_O and TFdi < 10% suggest low inspiratory muscles activity and thus low respiratory drive [4]. However, at presence of muscles weakness the limitation of these indices should be considered. It is of interest to note that P0.1 may be valid even in moderate to severe respiratory muscles weakness. It has been shown in an animal model of severe inspiratory muscles weakness, that P0.1 still increases reliably with increasing PaCO_2_, implying that the initial part of muscle contraction is relatively spared [81].

### Consequences of low drive

Now this patient is at risk of diaphragmatic atrophy. Indeed, it has been shown in animals that 12–18 h of PS, with a level of assist that caused diaphragmatic relaxation after triggering, resulted in diaphragmatic atrophy and contractile dysfunction [82]. Zambon et al. demonstrated in critically ill patients that there is a linear relationship between the level of PS and diaphragmatic atrophy rate [83]. Finally, Goligher et al. found that diaphragm atrophy is associated with a poor outcome [75]. Additionally, low respiratory drive is a risk factor of patient–ventilator dyssynchrony, mainly of the type of ineffective efforts [84, 85], which may contribute to poor outcome [86].

Further increase in PS level moves the supported ventilation curve to lower PaCO_2_ and when the intersection point is at PaCO_2_ lower than apneic threshold repetitive apneas occur, and respiratory drive is hover around zero [87, 88] (Fig. 6B). PaCO_2_ is close to apneic threshold. Non-steady state exists since the occurrence of apnea prevents PaCO_2_ to decrease considerably below the apneic threshold and reach the steady state. V’_E_ oscillates between zero to approximately 12 l/min. In addition to diaphragm atrophy, the patient is now at risk of poor sleep quality due to microarousals occurring at the end of each apneic episode. These microarousals result in severe sleep fragmentation and very low levels of deep sleep (sleep deprivation), further compromising the already poor sleep quality in these patients [89]. It is of interest to note that poor sleep quality is a risk factor for adverse short and long-term outcomes [90, 91]. The diaphragm may be also affected since it has been demonstrated that even one night of sleep deprivation in healthy individuals with normal function of the diaphragm may decrease inspiratory endurance due to reduction of cortical contribution to the respiratory centers output [15]. Finally, since the usual health care personnel response to apneas is to switch to control mechanical ventilation, unnecessary prolongation of mechanical ventilation is also a risk.

**Fig. 7:**
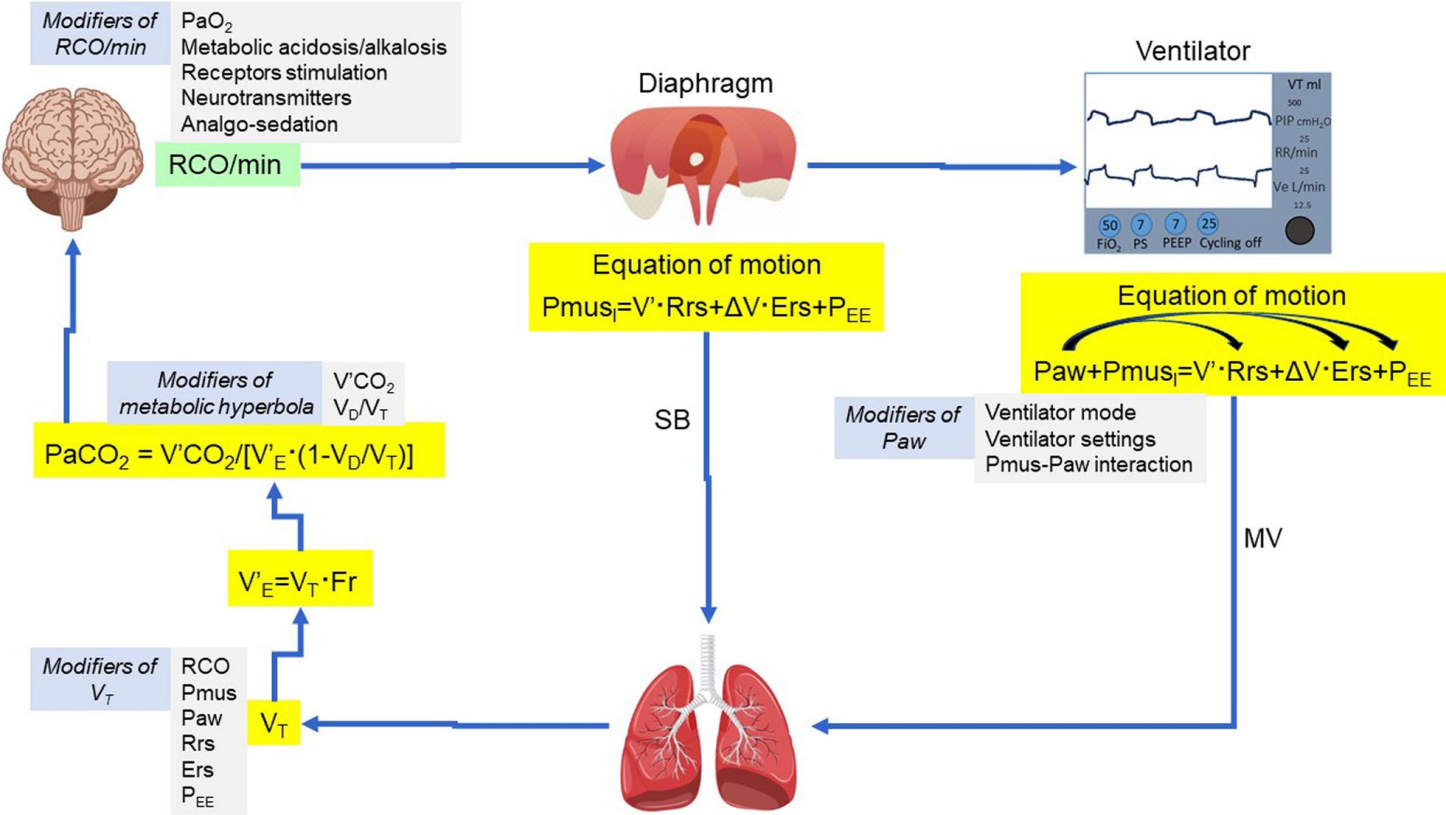
Determinants of brain curve (RCO/min/PaCO_2_), ventilation curve (V’_E_/PaCO_2_) and metabolic hyperbola during unsupported spontaneous breathing (SB) and mechanical ventilation (MV). MV modifies the equation of motion by applying pressure (Paw) to the lungs, which acts in conjunction with the pressure generated by the inspiratory muscles (Pmus_I_). During mechanical ventilation respiratory rate (Fr) may differ from the frequency of the electrical bursts (outputs) due to patient–ventilator dyssynchrony (i.e., ineffective efforts). Paw may change (curved arrows) Ers (recruitment/derecruitment/overdistension), Rrs (airway opening/closure) and P_EE_ (dynamic hyperinflation). Notice that tidal volume (V_T_) depends on a complex interaction of variables (Modifiers) determining brain curve, ventilation curve and metabolic hyperbola. RCO: respiratory centers output; Pmus: respiratory muscles pressure (inspiratory and expiratory); Ers: respiratory system elastance; Rrs: respiratory system resistance; P_EE_: elastic recoil pressure of respiratory system at end-expiration; V’: flow; ΔV: volume above end-expiratory lung volume; V’_E_: minute ventilation; V’CO_2_: CO_2_ production; V_D_/V_T_: physiological dead space to tidal volume ratio; PaCO_2_: partial pressure of arterial CO_2_; PaO_2_: partial pressure of arterial O_2_

In the example provided above, we focus on a patient with pneumonia who developed ARDS. Similar reasoning should be applied to other diseases that affect the brain curve, ventilation curve, and metabolic hyperbola [6, 35] (Fig. 7). For instance, this analysis demonstrated, contrary to general belief [92] that in patients with pulmonary arterial hypertension or chronic thromboembolic pulmonary hypertension the respiratory system is the main determinant of exercise limitation, with the cardiovascular system being an indirect contributor [6].

## Conclusion

Our analysis suggests that abnormalities in respiratory drive result from alterations in the brain curve, ventilation curve, and metabolic hyperbola. Considering the significant risks associated with both low and high respiratory drive, it is imperative to address and manage these abnormalities in all three curves. However, this task is complex, due to the significant interaction among the various factors that determine the curves (Fig. 7). In this process, it is important to recognize that respiratory drive can be increased by factors that: (1) impair the inspiratory flow-generation pathway (e.g., respiratory system mechanics derangements, dynamic hyperinflation, neuromuscular weakness) [35]; (2) increase the brain CO_2_ sensitivity (e.g., metabolic acidosis, hypoxemia, receptors stimulation) [41, 44]; and (3) shift the metabolic hyperbola upward (e.g., increases in V’CO_2_ and/or V_D_/V_T_) [39, 50–52]. Conversely, respiratory drive can be decreased by interventions/therapy that (1) reduce brain CO_2_ sensitivity (e.g., sedation, correction of metabolic acidosis or hypoxemia, metabolic alkalosis) [31, 40]; (2) restore the integrity of the pathway from the respiratory centers to tidal volume generation (e.g., mechanical ventilation, mode of support, titration of ventilator settings, improvements in respiratory system mechanics and neuromuscular weakness) [80, 93, 94], and (3) shift the metabolic hyperbola downward (e.g., decreases in V’CO_2_ or V_D_/V_T_) [39, 58]. By considering all factors that contribute to each of these three curves and employing inductive reasoning to understand their interactions, respiratory drive can be assessed at the bedside, facilitating a more informed decision-making process.

## Supplementary Information

**Additional file 1: Figure S1.** Normal (Health). Intact inspiratory flow-generation pathway. **Figure S2.** Neuromuscular weakness. **Figure S3.** Dynamic hyperinflation in a patient exhibiting flow limitation during passive expiration.

## Data Availability

Not applicable.

## Abbreviations

PaCO_2_: Arterial partial pressure of CO_2_
PaO_2_: Arterial partial pressure of O_2_
RCO: Respiratory centers output per breath
RCO/min: Respiratory centers output per minute
V’_E_: Minute ventilation
FRC: Functional residual capacity
V_T_: Tidal volume
RCO_I_: Respiratory centers output to inspiratory muscles
RCO_E_: Respiratory centers output to expiratory muscles
T_Im_: Mechanical inflation time
V’CO_2_: CO_2_ production
V_D_: Physiological dead space
V’/Q’: Ventilation–perfusion ratio
P-SILI: Patient self-inflicted lung injury
EAdi: Electrical activity of the diaphragm
Pdi: Trans-diaphragmatic pressure
Pmus_sw_: Respiratory muscle pressure swings
Pes: Esophageal pressure
P0.1: Airway occlusion pressure during the first 100 ms of inspiration
P_occl_: Absolute drop in airway pressure during a whole breath occlusion
TFdi: Thickening fraction of the diaphragm
ICU: Intensive Care Unit
PS: Pressure support
ARDS: Acute respiratory distress syndrome
