# Supplementary material for: Respiratory drive: a journey from health to disease
Source: J Intensive Care. 2024 Apr 22;12:15. doi: 10.1186/s40560-024-00731-5 (PMC11636889; doi:10.1186/s40560-024-00731-5)
Supplement: Supplementary file 1 — Additional file 1: Figure S1. Normal (Health). Intact inspiratory flow-generation pathway. Figure S2. Neuromuscular weakness. Figure S3. Dynamic hyperinflation in a patient exhibiting flow limitation during passive expiration. [file 40560_2024_731_MOESM1_ESM.docx]

**Additional file**

**Respiratory drive – A journey from health to disease**

Dimitrios Georgopoulos^1^, MD, PhD, Maria Bolaki^2^, MD, PhD, Vaia Stamatopoulou^3^

and Evangelia Akoumianaki^1,2^, MD, PhD

^1^Medical School, University of Crete, Heraklion, Crete, Greece. ^2^Department of Intensive Care Medicine, University Hospital of Heraklion, Heraklion, Crete, Greece. ^3^Department of Pulmonary Medicine, University Hospital of Heraklion, Heraklion, Crete, Greece

**Inspiratory flow-generation pathway in health and disease**

Figure S1: Normal (Health). Intact inspiratory flow-generation pathway


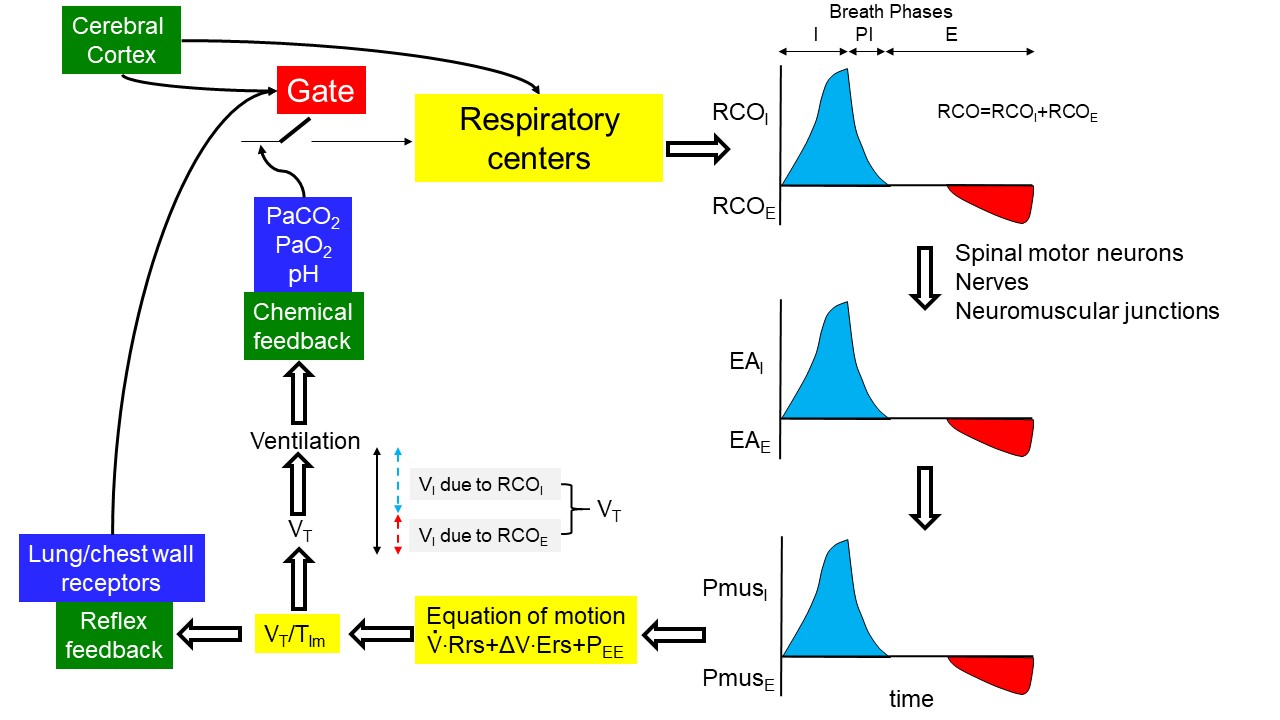


The inspiratory flow-generation pathway and the feedback mechanisms affecting it, in a normal subject during passive (no expiratory muscles activity) and active (expiratory muscles activity) expiration. For simplicity and demonstration purpose, RCO_I_ always begins when expiratory muscles activity ceases. Assuming that Pmus_E_ is able to lower lung volume below FRC (negative P_EE_), rapid relaxation of expiratory muscles (rapid decrease in Pmus_E_) passively generates inspiratory flow. When Pmus_E_ decreases to zero, FRC is reached. At this point Pmus_I_ increases and actively generates inspiratory flow. Notice, compared to passive expiration, the higher V_T_ with active expiration, which corresponds to higher RCO during the whole breath (respiratory drive).

Gate; The effects of afferent signals (inputs) on respiratory centers vary, depending on the breath phases (inspiratory, post-inspiratory, expiratory). RCO; Total respiratory centers output during the breath (respiratory drive). RCO_I_, RCO_E_; Respiratory centers output to inspiratory and expiratory muscles, respectively. EA_I_, EA_E_; electrical activity of inspiratory and expiratory muscles, respectively. Pmus_I_, Pmus_E_; Pressure generated by inspiratory and expiratory muscles, respectively. P_EE_; elastic recoil pressure of respiratory system at end expiration (zero at FRC, and positive and negative at volume above and below FRC, respectively). Ers; respiratory system elastance. Rrs; respiratory system resistance. ΔV; volume above end-expiratory lung volume. V_T_; tidal volume. V_I_; Inspired volume. Blue areas; RCO_I_, EA_I_ and Pmus_I_. Red areas; RCO_E_, EA_E_ and Pmus_E_. I, PI, E; Inspiratory, post inspiratory and expiratory phases, respectively.

Figure S2: Neuromuscular weakness


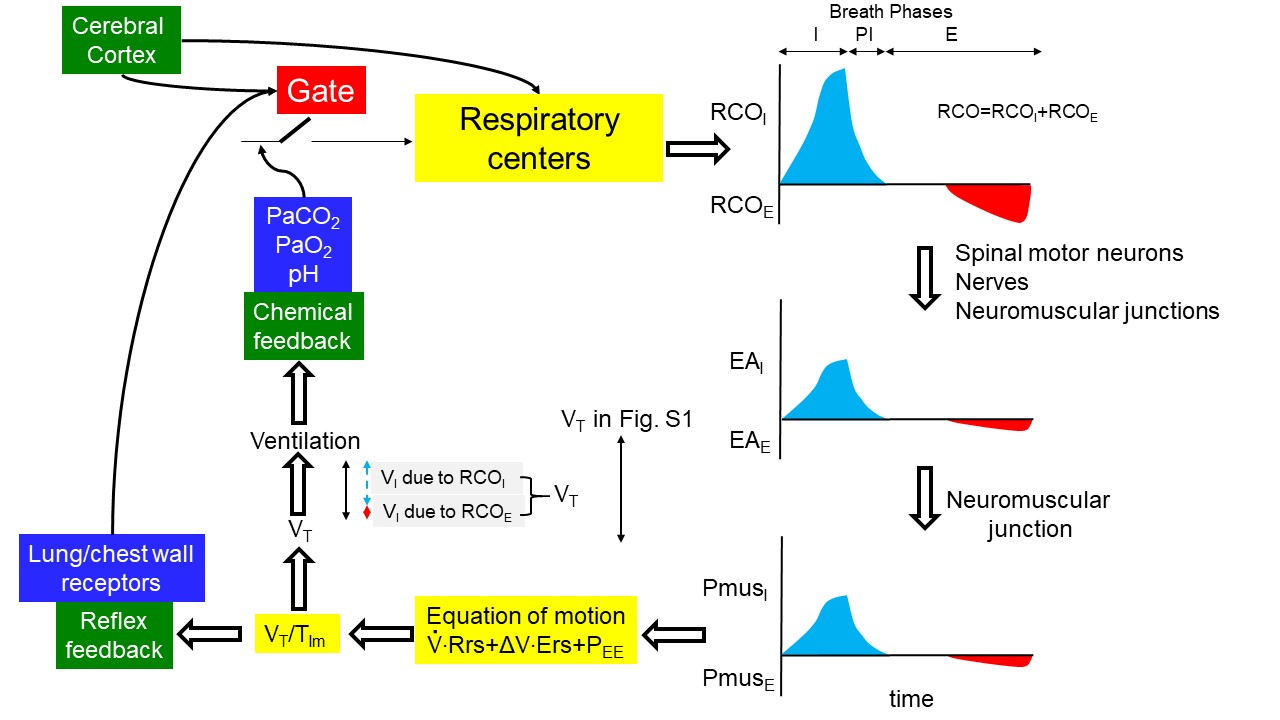


Figure S2: The inspiratory flow-generation pathway and the feedback mechanisms affecting it, in a simulated patient with neuromuscular weakness during passive (no expiratory muscles activity) and active (expiratory muscles activity) expiration. For simplicity and demonstration purpose, RCO_I_ starts when RCO_E_ ceases at FRC (P_EE_=0). Notice the discrepancy between RCO_I_ (blue area) and EA_I_ and Pmus_I_ (blue dotted areas) and between RCO_E_ (red area) and EA_E_ and Pmus_E_ (red dotted areas). V_T_/T_Im_ does not correspond to total RCO (respiratory drive). Assuming that Pmus_E_ is able to lower lung volume below FRC (negative P_EE_), rapid relaxation of expiratory muscles (rapid decrease in Pmus_E_) passively generates inspiratory flow. When Pmus_E_ decreases to zero, FRC is reached and Pmus_I_ increases and actively generates inspiratory flow. Notice, compared to passive expiration, the higher V_T_ with active expiration, which corresponds to higher RCO during the whole breath (respiratory drive).

Gate; The effects of afferent signals (inputs) on respiratory centers vary, depending on the breath phases (inspiratory, post-inspiratory, expiratory). RCO; Total respiratory centers output during the breath (respiratory drive). RCO_I_, RCO_E_; Respiratory centers output to inspiratory and expiratory muscles. EA_I_, EA_E_; electrical activity of inspiratory and expiratory muscles, respectively. Pmus_I_, Pmus_E_; Pressure generated by inspiratory and expiratory muscles, respectively. P_EE_; elastic recoil pressure of respiratory system at end expiration (zero at FRC, and positive and negative at volume above and below FRC, respectively). Ers; respiratory system elastance. Rrs; respiratory system resistance. ΔV; volume above end-expiratory lung volume. V_T_; tidal volume. V_I_; Inspired volume. Blue areas; RCO_I_, EA_I_ and Pmus_I_. Red areas; RCO_E_, EA_E_ and Pmus_E_.

Figure S3: Dynamic hyperinflation in a patient exhibiting flow limitation during passive expiration


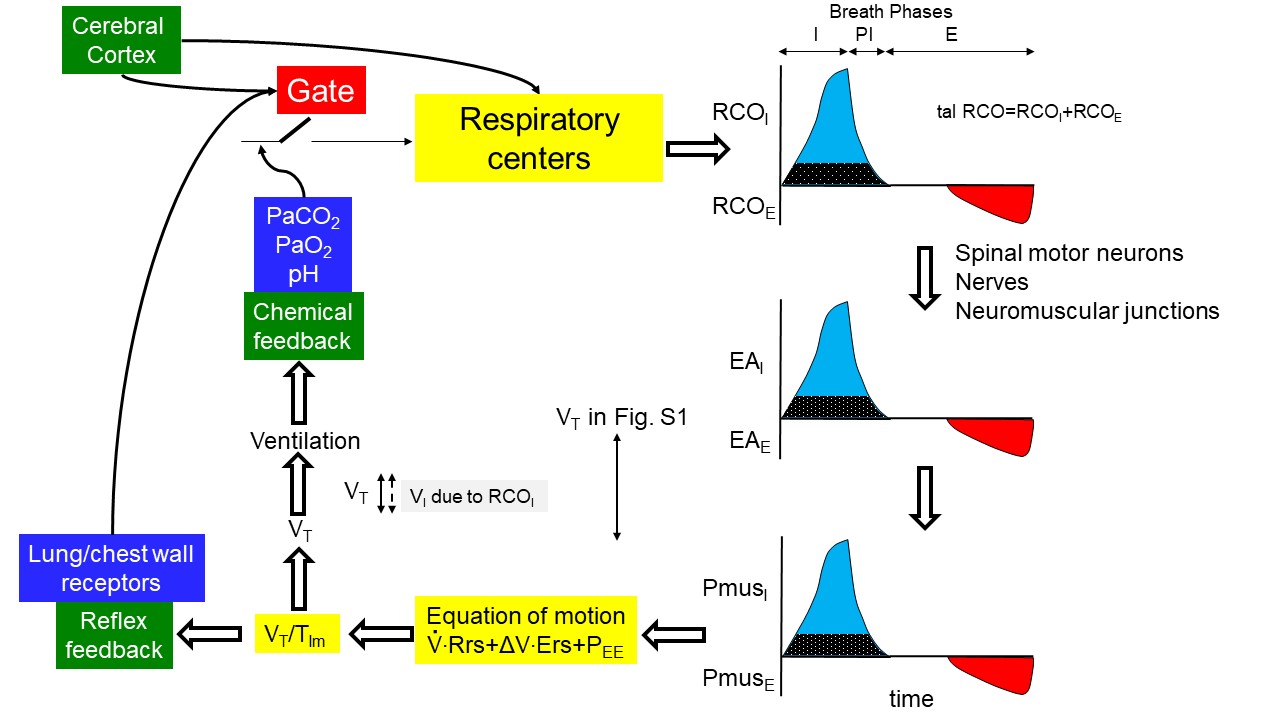


The inspiratory flow-generation pathway and the feedback mechanisms affecting it, in a simulated patient with chronic obstructive lung disease (increased Rrs), exhibiting dynamic hyperinflation (at the end of expiration P_EE_ is positive) and flow limitation during passive (no expiratory muscles activity) and active (expiratory muscles activity) expiration. For simplicity and demonstration purpose, RCO_I_ starts when RCO_E_ ceases. During passive expiration lung volume at the end of expiration, is above FRC, where P_EE_ is positive (dynamic hyperinflation). RCO_I_ must first increase EA_I_ and Pmus_I_ in order to overcome the positive P_EE_ (black dotted areas) and then to generate inspiratory flow (blue areas). During active breathing because of flow limitation Pmus_E_ (red area) is unable to increase expiratory flow above that achieved passively and therefore to decrease end-expiratory lung volume. Thus, with and without active expiration P_EE_ remains constant (positive). The contribution of expiratory muscle activity to V_T_ is nil. V_T_ is low because a portion of total RCO is able to generate inspiratory flow (blue area). Finally, the resistive pressure due to inspiratory flow is increased because of high Rrs (Pres=V’xRrs) and thus, V_T_ is further constrained. V_T_/T_Im_ does not correspond to total RCO (respiratory drive).

Gate; The effects of afferent signals (inputs) on respiratory centers vary, depending on the breath phases (inspiratory, post-inspiratory, expiratory). RCO; Total respiratory centers output during the breath (respiratory drive). RCO_I_, RCO_E_; Respiratory centers output to inspiratory and expiratory muscles. EA_I_, EA_E_; electrical activity of inspiratory and expiratory muscles, respectively. Pmus_I_, Pmus_E_; Pressure generated by inspiratory and expiratory muscles, respectively. P_EE_; elastic recoil pressure of respiratory system at end expiration (zero at FRC, and positive and negative at volume above and below FRC, respectively). Ers; respiratory system elastance. Rrs; respiratory system resistance. ΔV; volume above end-expiratory lung volume. V_T_; tidal volume. V_I_; Inspired volume. Blue areas; RCO_I_, EA_I_ and Pmus_I_. Red areas; RCO_E_, EA_E_ and Pmus_E_.
